# Random-forest algorithm based biomarkers in predicting prognosis in the patients with hepatocellular carcinoma

**DOI:** 10.1186/s12935-020-01274-z

**Published:** 2020-06-17

**Authors:** Lingyun Guo, Zhenjiang Wang, Yuanyuan Du, Jie Mao, Junqiang Zhang, Zeyuan Yu, Jiwu Guo, Jun Zhao, Huinian Zhou, Haitao Wang, Yanmei Gu, Yumin Li

**Affiliations:** 1 Department of General Surgery, Lanzhou University Second Hospital, Lanzhou, 730030 Gansu China; 2 Lanzhou University Second Hospital, Lanzhou, 730030 Gansu China; 3 Key Laboratory of Digestive System Tumors of Gansu Province, Lanzhou, 730030 Gansu China; 4 The Second Clinical Medical College of Lanzhou University, Lanzhou, 730030 Gansu China

**Keywords:** Hepatocellular carcinoma, Random-forest algorithm, TCGA, SPC25, NUF2, MCM2, BLM and AURKA

## Abstract

**Background:**

Hepatocellular carcinoma (HCC) one of the most common digestive system tumors, threatens the tens of thousands of people with high morbidity and mortality world widely. The purpose of our study was to investigate the related genes of HCC and discover their potential abilities to predict the prognosis of the patients.

**Methods:**

We obtained RNA sequencing data of HCC from The Cancer Genome Atlas (TCGA) database and performed analysis on protein coding genes. Differentially expressed genes (DEGs) were selected. Gene Ontology (GO) and Kyoto Encyclopedia of Genes and Genomes (KEGG) pathway enrichment were conducted to discover biological functions of DEGs. Protein and protein interaction (PPI) was performed to investigate hub genes. In addition, a method of supervised machine learning, recursive feature elimination (RFE) based on random forest (RF) classifier, was used to screen for significant biomarkers. And the basic experiment was conducted by lab, we constructe a clinical patients’ database, and obtained the data and results of immunohistochemistry.

**Results:**

We identified five biomarkers with significantly high expression to predict survival risk of the HCC patients. These prognostic biomarkers included SPC25, NUF2, MCM2, BLM and AURKA. We also defined a risk score model with these biomarkers to identify the patients who is in high risk. In our single-center experiment, 95 pairs of clinical samples were used to explore the expression levels of NUF2 and BLM in HCC. Immunohistochemical staining results showed that NUF2 and BLM were significantly up-regulated in immunohistochemical staining. High expression levels of NUF2 and BLM indicated poor prognosis.

**Conclusion:**

Our investigation provided novel prognostic biomarkers and model in HCC and aimed to improve the understanding of HCC. In the results obtained, we also conducted a part of experiments to verify the theory described earlier, The experimental results did verify our theory.

## Background

Hepatocellular carcinoma (HCC) is considered to be the most common liver cancer in the world, ranking fifth in men and seventh in women [1]. The development of liver cancer is highly correlated with the infection of hepatitis B virus (HBV) and/or hepatitis C virus (HCV) [2]. Surgical resection is the main treatment for most cases of liver cancer (HCC) and only 30 to 40% of patients with liver cancer can be treated after diagnosis by surgical resection [3]. Therefore, it is important to find an effective and reliable diagnosis of liver cancer that can significantly improve the diagnosis of liver cancer patients.

Carcinogenesis is a multi-step process, which is a change caused by signal pathways triggered by multiple genes, which transforms normal cells into malignant cells [4, 5]. The molecular mechanism of the occurrence and development of HCC are unclear. However, it is considered that, at the beginning of the preneoplastic stage, genetic changes in a few genes and chromosomal loci will slowly accelerate and enhance the transition of hepatocytes from atypical hyperplasia to liver cancer [6]. With the development of Next Generation Sequencing (NGS) technologies, we have located key carcinogenic genes and related oncogenic signaling pathways that play a pivotal role in the initiation and progression of HCC. However, despite the availability of a large amount of public genetic information, effective diagnostic methods are needed to predict the prognosis of HCC.

With a series of changes in biological processes, such as immune regulation, cell cycle, angiogenesis, healing, and auto-swallowing, genetic mutations contribute significantly to tumor formation [7–11]. Differentially expressed genes (DEGs) are involved in changes in signal routing and biological processes during tumor formation. Tracks were not independent in their function, which are linked between tracks. Interfering genes revealed by related pathways are potential biomarkers and therapeutic targets for cancer. Important information about liver cancer can be found in these intersecting genes.

In this study, the HCC gene expression profile data was downloaded from the public database to determine a linear risk score as a survival prediction model based on the HCC interference genes and for identify biomarkers that predict the risk of survival for patients with liver cancer.

## Materials and methods

### RNA-seq transcriptome data of samples

We downloaded the RNA-sequencing (RNA-seq) expression profiles of HCC from TCGA database (https://cancergenome.nih.gov/, up to Nov.03, 2016), involving 423 samples. These samples contained 373 HCC tumor tissues and 50 normal liver tissues, which were publicly available and open-access. The clinical data of HCC patients were also obtained from TCGA and 369 patients with complete survival data were enrolled in further survival analysis. Data acquired from TCGA database were carried out by the Illumina HiSeq Systems. Data format of sequencing is Counts files.

### Gene reannotation

RNA-sequencing data got from the TCGA contained multiple types of RNA, including long non-coding RNA (lncRNA), protein coding genes and pseudogenes. The transcriptome data was reannotated to identify the gene symbols based on annotation file (Homo_sapiens.GRCh38.87.chr.gtf) downloaded from Ensemble gene browser (http://www.ensembl.org/). Only the protein coding genes were selected during the annotation. Others were filtered in this step.

### Identification of differentially expressed genes (DEGS)

The Location of DEGs was the first step in our research. It played a crucial role in the studying internal mechanism in HCC [12].The identification was conducted by R/edgeR, obtained from an open-source Bioinformatics project, Bioconductor (http://www.bioconductor.org/) [13, 14]. The negative binomial distributions is the key foundation of the package, also involving empirical Bayes estimation, exact tests, generalized linear models (GLM) and quasi-likelihood tests. LogFC ≥ 2.0 or logFC ≤ −2.0 associated with the P value < 0.01 were selected as the statistically significant difference.

### Gene Ontology and KEGG pathway enrichment

The analysis of Gene Ontology (GO) and the Kyoto Encyclopedia of Genes and Genomes (KEGG) pathway enrichment is an essential aspect of Bioinformatics to reveal the biological functions and molecular mechanisms of DEGs [15, 16]. DAVID database (https://david.ncifcrf.gov/) is designed as a web tool which contains the relevant biological annotation. We revealed the biology function and pathways of DEGs with DAVID. In this step, the false discovery rate (FDR) < 0.1 was considered as significance for filtering the GO terms and KEGG pathways.

### Protein–protein interaction (PPI) network of DEGS

Search Tool for the Retrieval of Interacting Genes (STRING) database (http://www.string-db.org/) is an important Bioinformatics tool for determining the relationship between genes [17]. We performed the PPI analysis in order to promote our understanding of undetected connection underlying the DEGs. Here, we choose only the experimentally validated PPI links with its combined score > 0.7 to enhance the reliability. Nodes with no links with others were discarded. We defined a C-score (Connection score) to measure the hub degree for every node. The genes with a high C-score had the potential to be the biomarkers. We screened the genes for their C-score ≥ 5 as the significance.

### Significant biomarkers selection

The expression of significant hub genes got from PPI analysis was log2 scale. For the prognostic signature analysis, the 369 HCC samples that contained complete clinical data were assigned into groups of good or poor prognosis according to the 5 years survival (expected survival time > 5 or < 5 years). Recursive Feature Elimination (RFE) based on Random Forest (RF) classifier, a method of supervised Machine Learning, was conducted to identify the prognostic genes in survival [18, 19]. The prediction was examined by fivefolds cross-validation. We selected the best prognostic genes according to the accuracy of the RFE-RF predictor. Genes selected by RFE-RF were chosen as the candidate biomarkers. The selection was performed with R/Caret package.

### Survival model

The genes selected from RFE-RF were considered as the variables for survival analysis. We separated the 369 patients with complete clinical data into training (n = 239) and testing (n = 130) datasets randomly. To better investigate the performance of these genes in predicting survival, multivariate Cox regression model was conducted in the training dataset. The coefficients were used as the weight for genes’ expression to create a risk score model. Besides, samples were divided into two groups according to the median value of risk score model. Keplan-Meire (KM) method was performed to test the prognostic performance of the model. All the analysis was used with R/survival package.

### Patients’ information and tissue samples

Tissue samples were taken from patients who had undergone liver resections in the Second Hospital of Lanzhou University. All patients received liver resections from July 2012 to December 2014. None of the patients received preoperative chemotherapy and radiotherapy. All patients were followed until December 2018. Details of the clinical traits of all patients are shown in Tables 1 and 2. All groups were assessed and unidentified based on ethical criteria. The period of time between the operation and death or final result is defined as Legislation General survival (OS). Survivorship Disease (DFS) is regared as the period elapsed between performed surgery and tumor development.

**Table 1:** Correlation between NUF2 expressions with clinic-pathological characteristics of HCC

Clinicopathological variables	N	NUF2 expression		*P* value
		Low (37)	High (58)	
Sex				0.482
Male	81	31	50	
Female	14	6	8	
Age, years				0.533
< 50	48	19	29	
≥ 50	47	18	29	
AFP, ng/L				*< 0.001*
< 200	53	29	24	
≥ 200	42	8	34	
HBsAg				0.439
Negative	44	12	32	
Positive	51	25	26	
Tumor size, cm				0.075
≤ 5	49	23	26	
> 5	46	14	32	
Tumor nodule number				*0.001*
Solitary	57	30	27	
Multiple (≥ 2)	38	7	31	
Cancer embolus				*0.002*
Absence	62	31	31	
Presence	33	6	27	
TNM stage				*0.013*
Early (I & II)	52	26	26	
Late (III & IV)	43	11	32	
Differentiation grade				0.571
Well	67	26	41	
Poor	28	11	17	

*AFP* alpha fetoprotein, *HBsAg* hepatitis B surface antigen

**Table 2:** Correlation between BLM expressions with clinic-pathological characteristics of HCC

Clinicopathological variables	N	BLM expression		*P v*alue
		Low (55)	High (40)	
Sex				0.208
Male	81	45	36	
Female	14	10	4	
Age, years				0.171
< 50	48	25	23	
≥ 50	47	30	17	
AFP, ng/L				*0.022*
< 200	53	36	17	
≥ 200	42	19	23	
HBsAg				0.504
Negative	44	25	19	
Positive	51	30	21	
Tumor size, cm				*0.005*
≤ 5	49	35	14	
> 5	46	20	26	
Tumor nodule number				*0.01*
Solitary	57	39	18	
Multiple (≥ 2)	38	16	22	
Cancer embolus				0.567
Absence	62	36	16	
Presence	33	19	14	
TNM stage				*0.033*
Early (I & II)	52	35	17	
Late (III & IV)	43	20	23	
Differentiation grade				0.550
Well	67	39	28	
Poor	28	16	12	

*AFP* alpha fetoprotein, *HBsAg* hepatitis B surface antigen

### Immunohistochemical staining and antibodies

Tissue samples from 95 cases of hepatocellular carcinoma were used in formalin and paraffin embedded for NUF2 and BLM immunohistochemistry. NUF2 and BLM antibodies for staining of immunochemistry were obtained from ABCAM (ab230313 and ab62206). After defrost, moisture, and embolism, samples were mixed with NUF2’s primary immunoglobulin antibodies and BLM antibodies and then incubated at night at 4 (dilution ratio 1: 1000). Finally, all sections were evaluated by comparing the staining of each sample of cancer cells from the liver and the normal sample under a microscope. The positive cell score and color intensity determine the overall score. The degree of intensity of the coloration is as bellow: 0: no coloration. 1: slightly yellowish in the background, 2: yellow and brown. 3: Brown. The positive result of the cell is as follows: 0 degree: 0–5%; 1 degree: 6–25%; 2 degree: 26–50%; 3 degree: 51–75%; 4 degree: > 75%. The overall result of immunochemistry was calculated as a positive degree of x-cell staining intensity. The overall score is divided by four levels: 0 means negative (−), 1–4 defined as positive weakness (+), 5–8 stand for positive (++) and 9–12 regared strongly positive (+++).

### Statistical analysis

All data analyses were conducted with edgeR. Evaluation of patient samples were evaluated using Pierson correlation coefficients. Survival rate calculated using Cox proportional hazard model. Survival curves were calculated by the Kaplan–Meier method.

## Results

### Identification of DEGs

**Fig. 1:**
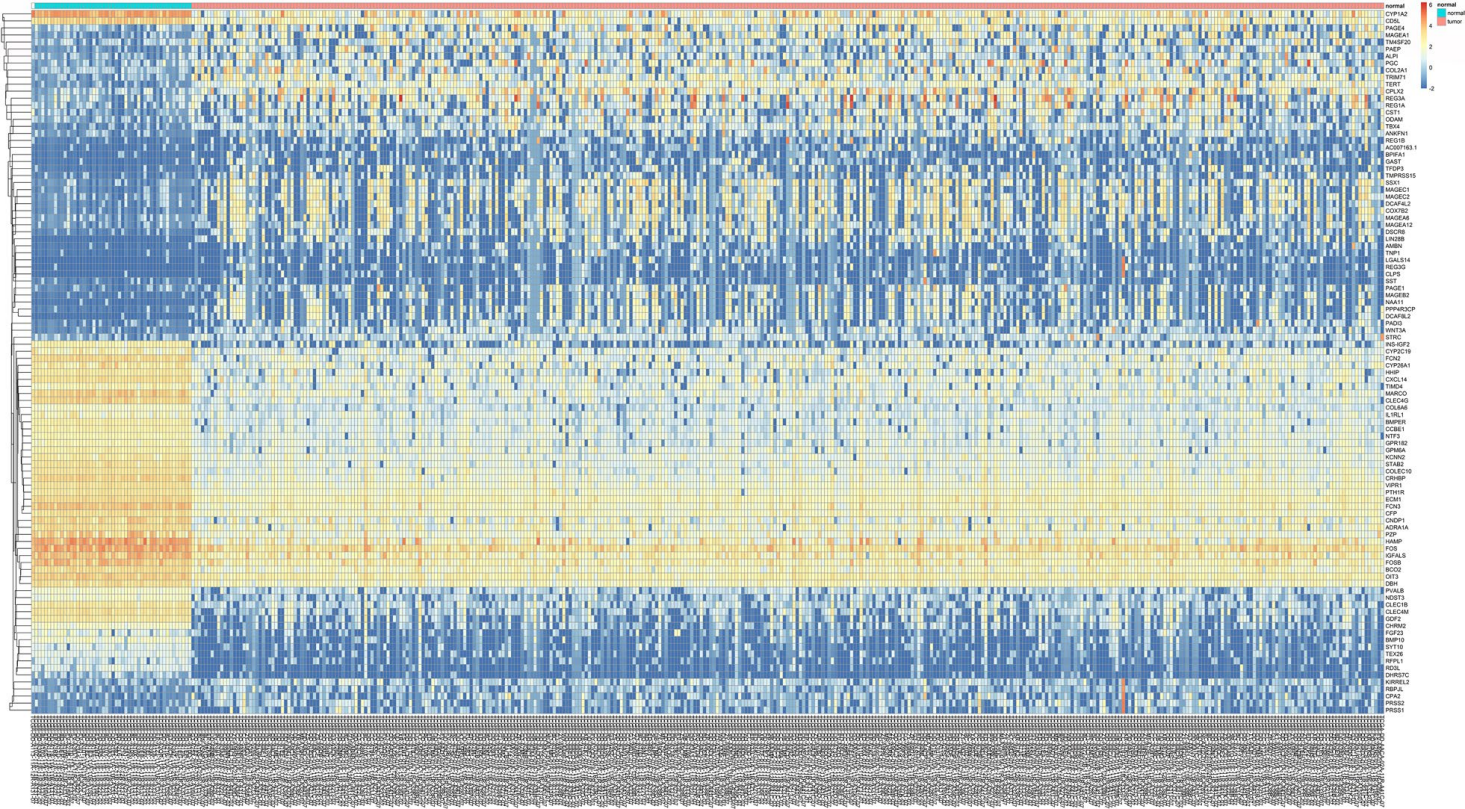
Heatmap of top 50 up-regulated and down-regulated genes with the highest LogFC

Identification of the DEGs in HCC samples demonstrated that there were 1844 up-regulated DEGS and 213 down-regulated DEGS, based on R/edgeR. The heat map of the DEGs (top 50 up-regulated and down-regulated genes according to the LogFC) is shown as an example (Fig. 1).

### GO and KEGG pathway enrichment

**Fig. 2:**
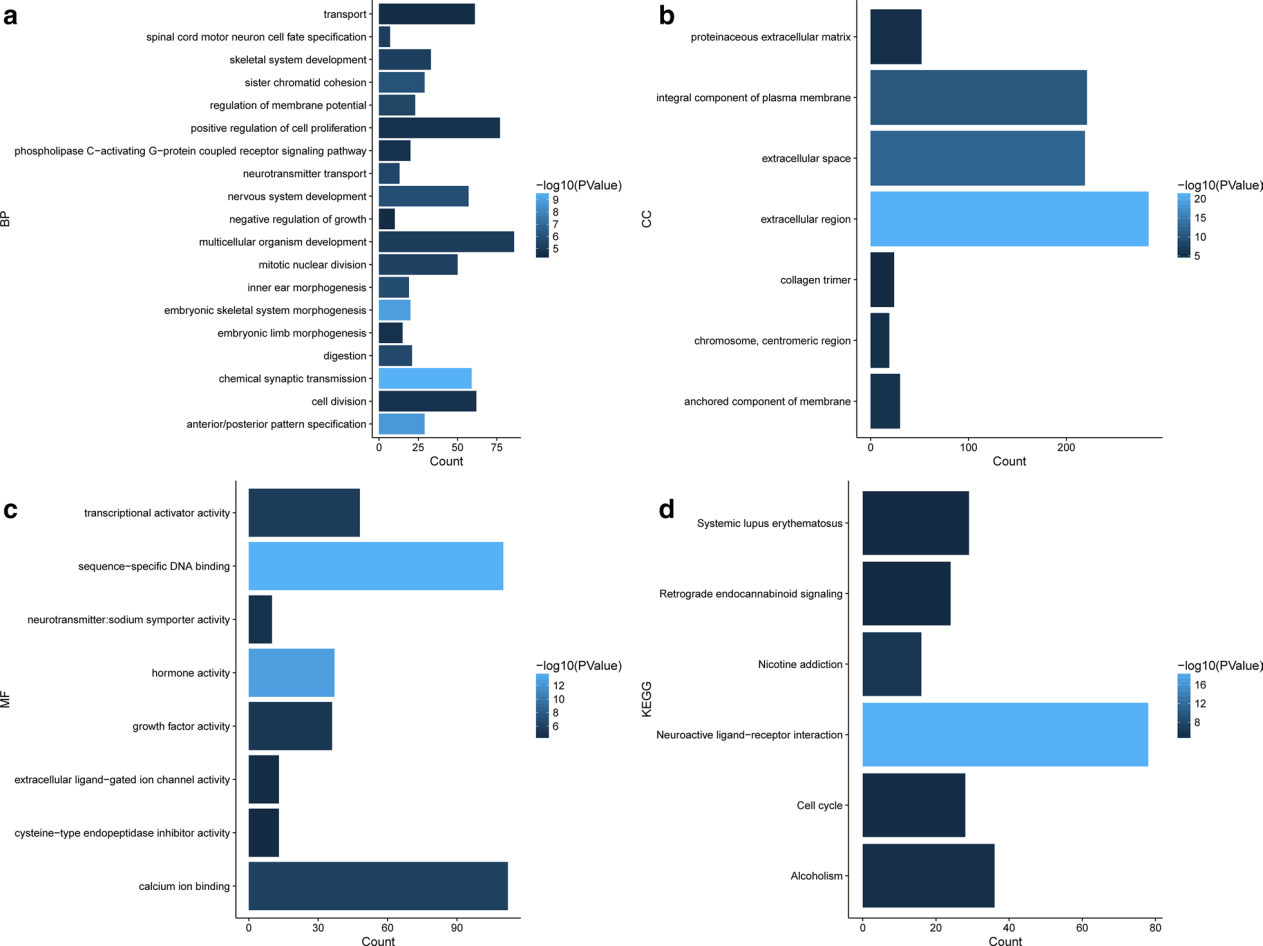
GO terms and KEGG pathways enrichment of DEGs with FDR < 0.1. (**a** Biological_processes, **b** Cellular_component, **c** Molecular_function, D. KEGG pathways)

The enriched GO terms were totally separated in three groups biological process (BP), cellular component (CC) and molecular function (MF). The result of GO enrichment showed DEGs participated in lots of significant biology processes, such as extracellular region, sequence-specific DNA binding, extracellular space and transcriptional activator activity. KEGG pathways enrichment demonstrated DEGs were mapped on the several important pathways, including cell cycle and neuroactive ligand-receptor interaction (Fig. 2).

### PPI analysis and biomarker selection

**Fig. 3:**
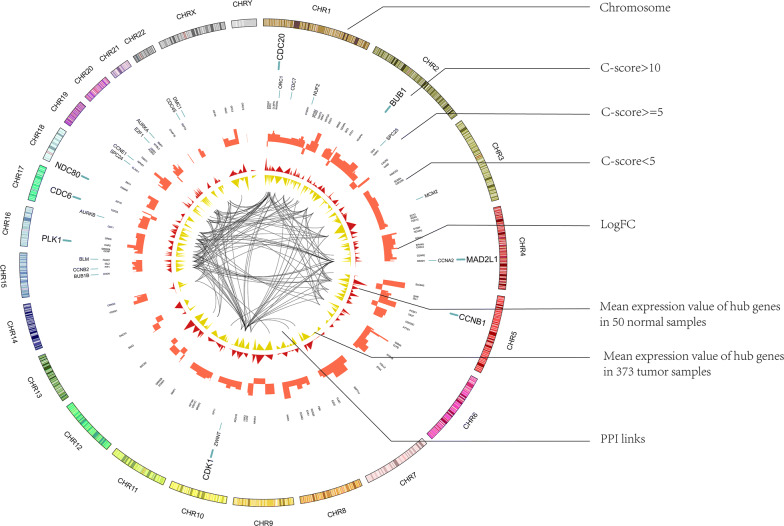
Circos Map of hub genes in PPI analysis, including 8 layers. From outside to inside: chromosome; hub genes with C-score > 10; hub genes with C-score ≥ 5; hub genes with C-score < 5; LogFC of hub genes; expression in normal tissues; expression in tumor tissues; PPI interaction links

**Fig. 4:**
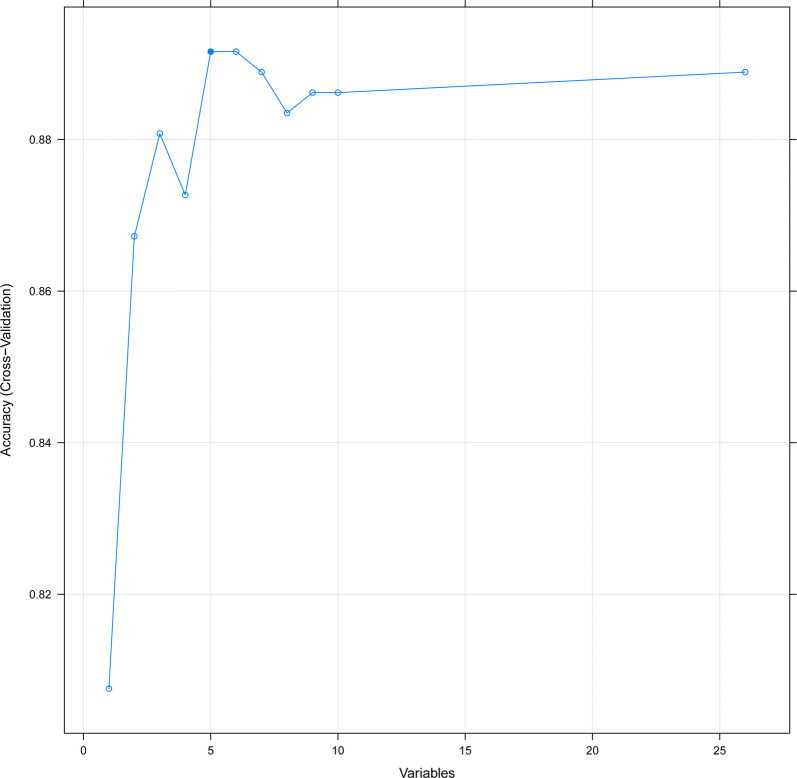
The relationship between variables and accuracy in RFE-RF predictor, with the fivefolds cross-validation

26 hub genes were screened from the PPI network for their hub degree ≥ 5. Among these hub genes, CDK1 showed the highest node degree, which was 22. We illustrated the Circos map of the hub genes to disclose their location on the chromosome and the links with others (Fig. 3). Besides, 5 genes including SPC25, MCM2, NUF2, AURKA and BLM were screened from the RFE-RF method. The accuracy of five candidate biomarkers for predicting prognosis reaches 0.89 (Fig. 4).

### Risk score survival model of 5 biomarkers

**Fig. 5:**
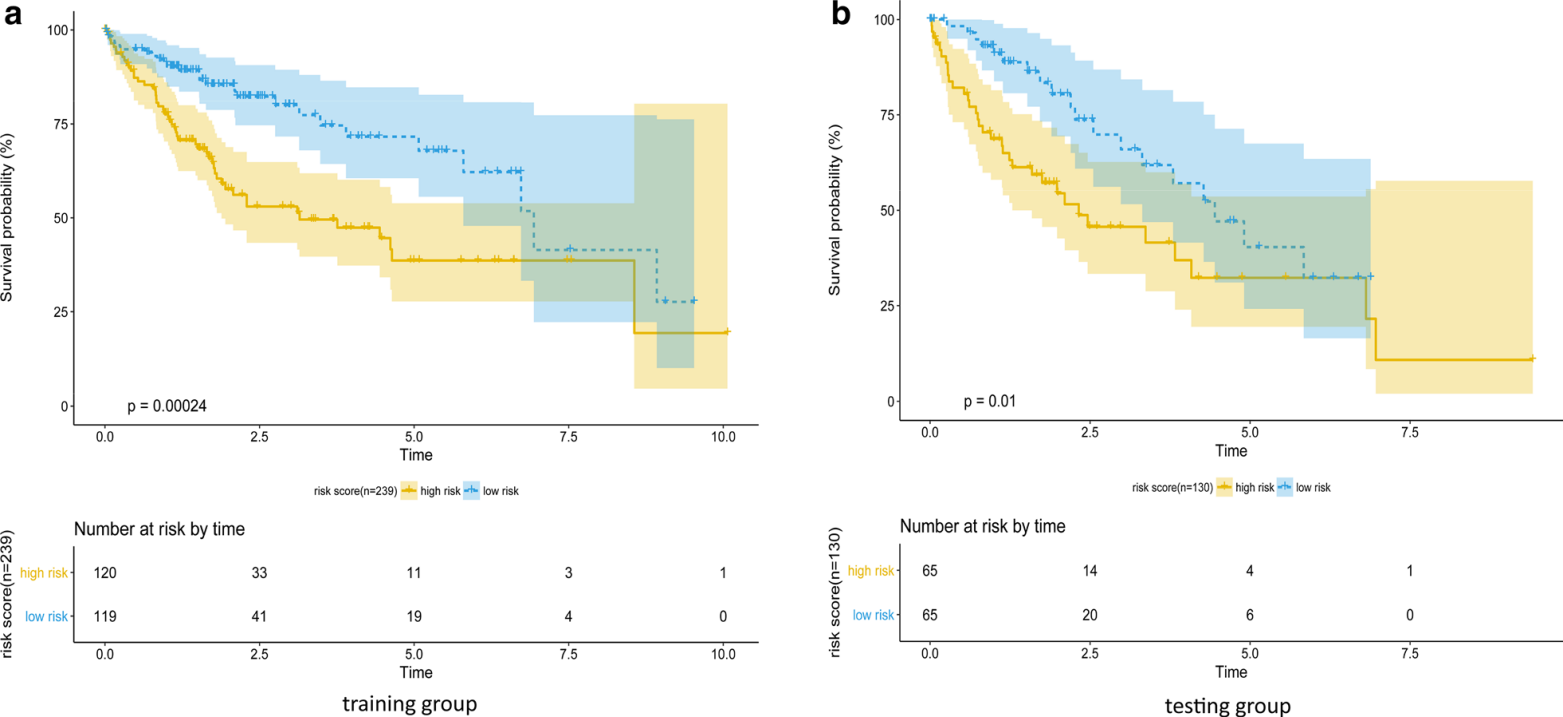
Performance of the risk score model in training (n = 239) and testing (n = 130) groups, examined by Keplan-meier method

**Fig. 6:**
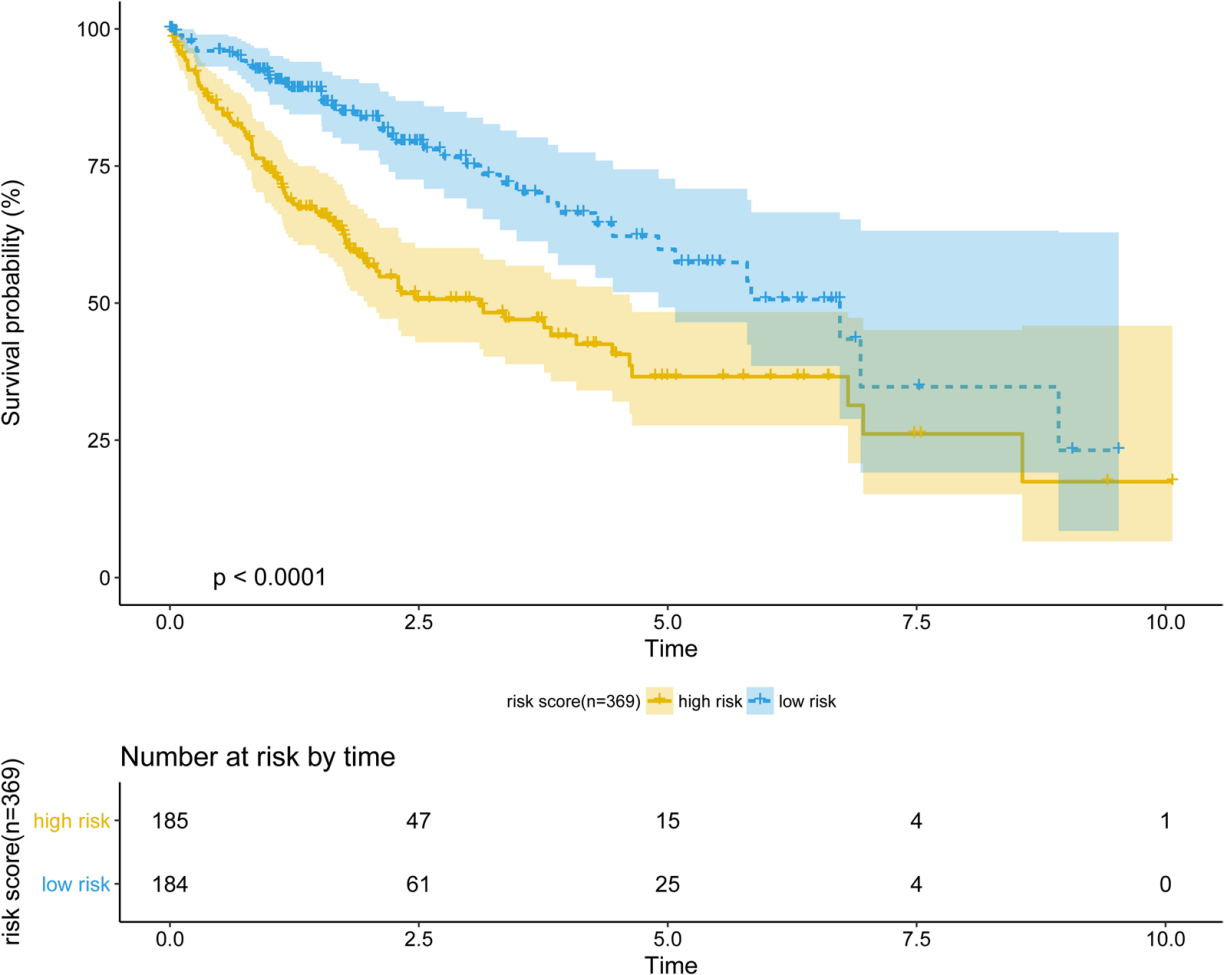
Performance of risk score model in full dataset (n = 369), examined by Keplan-meier method

**Fig. 7:**
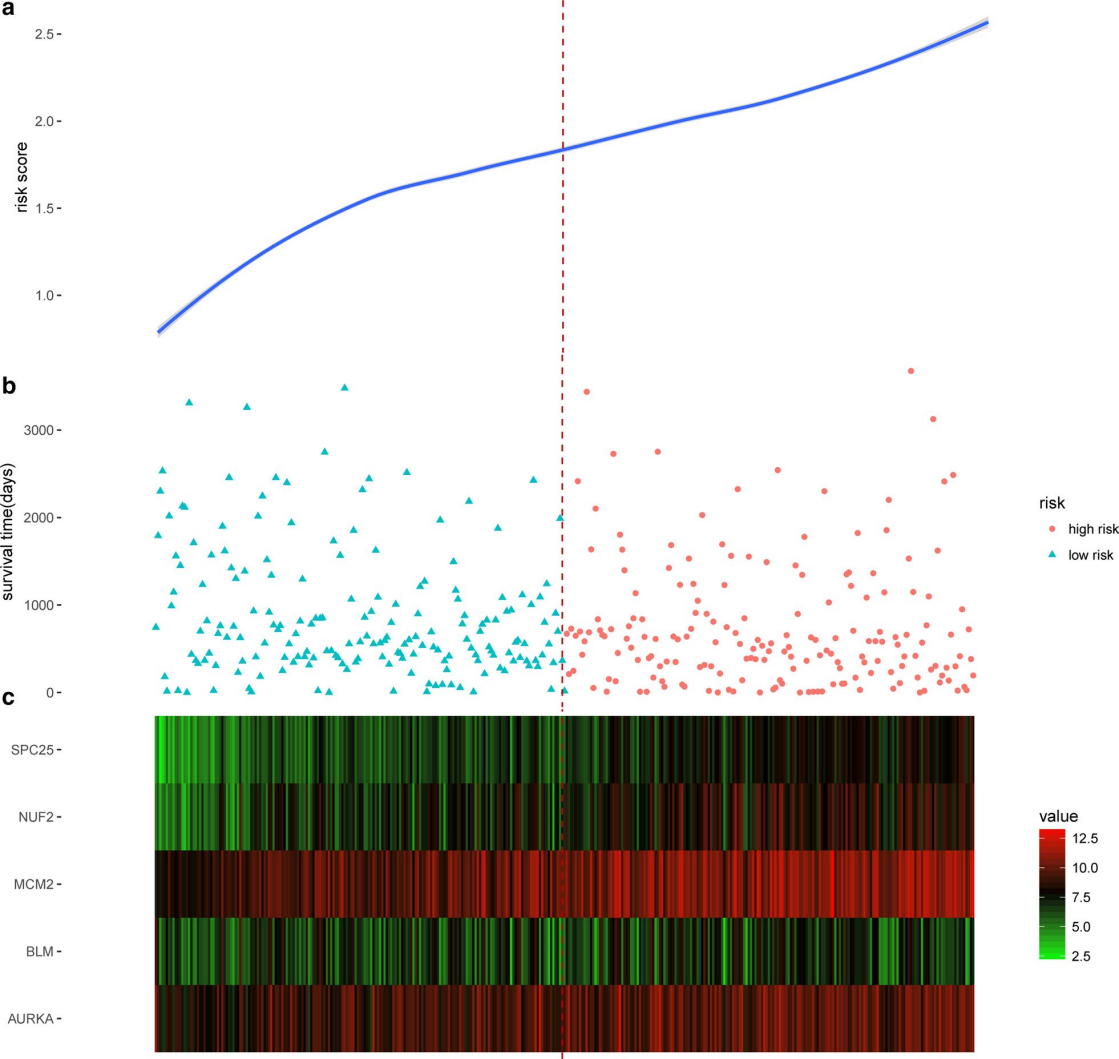
Performance validation of risk score model. **a** Risk score distribution, **b** survival time of the patients, sorted by risk score, **c** expression pattern of five prognostic biomarkers in 369 patients

The risk score model was carried out by multivariate Cox regression model. The coefficients of the result were used as the weight for each gene to create a risk score model. Risk score = (0.3497 × expression level of SPC25) + (0.0995 × expression level of MCM2) + (0.0327 × expression level of NUF2) + (0.0369 × expression level of AURKA) + (-0.3185 × expression level of BLM). The risk score model was examined in the testing group and full dataset with KM curve and P value (Figs. 5 and 6). The patients with higher risk scores had the worse survival compared with lower ones (Fig. 7). Risk score had the negative correlation with overall survival (OS). The analysis suggested risk score model can be considered as an independent clinical feature for OS of the patients with HCC.

### Overexpression of NUF2 and BLM imply poor survival in patients with HCC

**Fig. 8:**
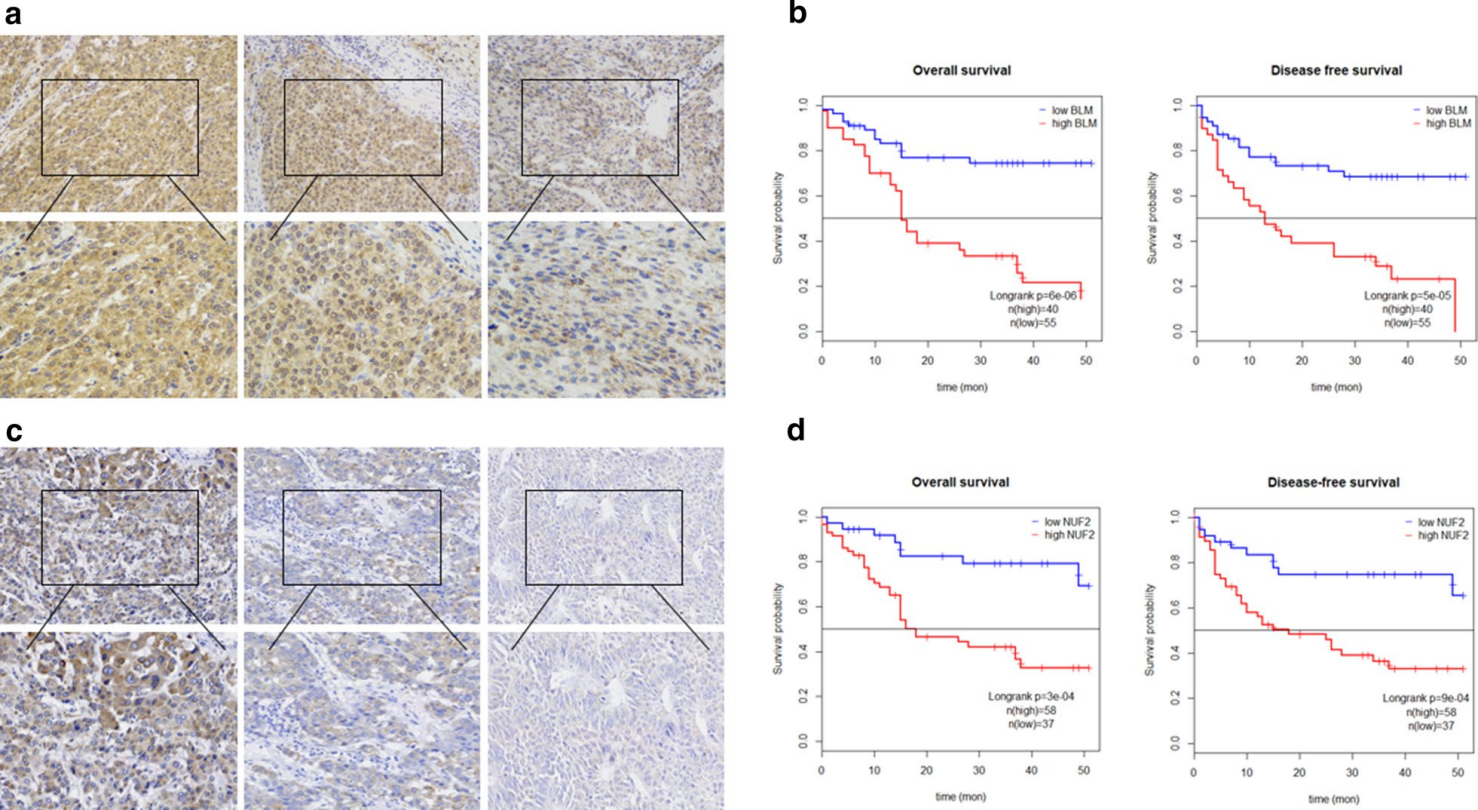
**a**, **c** NUF2 and BLM expressions are correlated with clinic-pathological features and poor prognosis. Immunohistochemical staining showed low NUF2 and BLM expressions in normal liver tissues and HCC tissues. The scales bars indicate 50 μm and 20 μm. **b**, **d** Overall survival and disease-free survival curves for HCC patient groups. All *P < 0.05, **P < 0.01

To ascertain the prognostic value of NUF2 and BLM, We performed a KM analysis, and all results are detailed in Fig. 8. Hyper Expression of NUF2 and BLM shown lowest OS and DFS. Next, Cox relative risk model is used to examine Whether NUF2 and BLM can become independent diagnostic influence factor for 95 patients suffering from liver cancer in our center. Results show poor prognosis in patients with high expression of NUF2 and BLM (Fig. 8). Multivariate analysis showed that the expression NUF2 and BLM (HR 2.35, 95% CI 1.06–6.11, P < 0.05) were independent predictors of the operating system. Therefore, we can think that nuf2 and BLM can provide independent prediction for liver cancer patients.

## Discussion

Although many gene products affecting liver cancer have been discovered, the molecular mechanisms underlying the occurrence and development of HCC are still unclear. Thereafter, it is useful to improve the diagnosis in patients with liver cancer by detecting the vital signs that contribute to the diagnosis and treatment of liver cancer [20]. These changes can control the global regulatory mechanisms that lead to collaboration between different metabolic pathways and different signals. Therefore, the cross-interaction genes examined from these associated pathways could be the major biomarkers of HCC.

In this study, HCC RNA expression profiles were downloaded for HCC and DEGs were examined. A number of 1844 regular genes were extracted from tumor samples and 213 regular genes were obtained from subcancerous liver tissue, which were assigned to a compact PPI network. Then select pivotal genes based on RFE-RF predictor accuracy and node degree in the PPI network [21]. A number of 100 pivotal genes were detected. KEGG analysis of the enrichment pathway implied that these pivotal genes were remarkably enriched in 22 pathways including pathways in the cell cycle and interaction between neuron receptor receptors, which were Reported to be ramarkbaly related to the occurrence of liver cancer [10, 22]. We then categorized 100 survival genes through random survival forests, the most important biomarkers are SPC25, MCM2, NUF2, AURKA and BLM.

Some studies have recently shown that unregulated SPC25 is associated with the carcinogenic process and malignant patterns of certain tumors. The regulation of SPC25 is found in colorectal and gastric cancers [23]. It acts as a gene systematically linked to liver cancer, associated with early recurrences after curative resection [24]. Many clinical parameters such as advanced tumor score, advanced stage, and poor prognosis in malignancies are highly related with MCM2 [25–27]. In addition, their study revealed that the cytoplasmic compound MCM2-gp70 associated with protein phosphatase 2A (PP2A) interferes with the PP2A-DNA-PK reaction and promotes apoptosis caused by DNA damage by activating p53 by DNA-PK [28]. NUF2 has been discovered to participate in cancerous tumors of many types of human tumors. Previous studies suggest that depletion of NUF2 by specific siRNAs inhibit proliferation and induce apoptosis in non-small cell and ovarian cancer cells [23, 29, 30]. Similarly, a reduction in NUF2 inhibited tumor growth caused by apoptosis in human tumor cells [31]. In addition, NUF2 played a key role in pancreatic cancer profiles by regulating RNA lnc RNA 339813 [32]. It has been shown that Aurka was involved in many cancers and was aneuploidy and genetic instability [33, 34]. The main functional partner proteins include inhibitors of MYCN, NFKBa, AKT1, RALA, P53 and BRCA1 [33, 35–40]. AURKA regulates the phosphorylation of these important carcinogenic proteins leading to their respective pathways. Other evidence of PLA, FAK and Src [41]. Bloom syndrome (Bloom’s Syndrome, BLM), a member of the Recase helase family, is one of the essential vaccines required for the metabolic processes of DNA, including recombination, redundancy, and repair of DNA. DNA. It is known that the level of BLM expression is regulated differently during cell cycle stages and is expressed at high levels in cancer cells. Since BLM abnormalities are associated with genome instability, evidence accumulates in various cancers [42–44]. Previous studies have also revealed that the role of BLM in p53 is binding in the Chk1 pathway [45].

## Conclusions

We collected a highly reliable database of hepatocellular carcinoma and used these datasets to build a survival prediction model based on the above 5 genes through multi-variable Cox regression. This risk score predicted patients at high risk of mortality independently. Immunohistochemical experiments were performed, and the results shown that NUF2 may play an pivotal role in promoting the occurrence and development of liver cancer, but the mechanism needs more research to demonstrate and and this is what we are doing. Our current work aims to provide the fresh methods for the clinical application of gene expression profiling in HCC, Especially in the future, this method will be applied to individualized prediction of disease and precision medicine, But the reliability and accuracy of this risk assessment must be verified through more forward-looking studies.

## Data Availability

The datasets generated and/or analysed during the current study are available in the TCGA repository. http://www.tcga.org

## Abbreviations

HCC: Hepatocellular carcinoma
TCGA: The Cancer Genome Atlas
DEGs: Differentially expressed genes
GO: Gene Ontology
KEGG: Kyoto Encyclopedia of Genes and Genomes
PPI: Protein and protein interaction
RFE: Recursive feature elimination
RF: Random forest
